# Exploring the relationship between lower-limb strength and neuromuscular activation in running: Insights from integrated EMG

**DOI:** 10.1016/j.isci.2026.115232

**Published:** 2026-03-11

**Authors:** Qin Zhang, Bas Van Hooren, Olivier Girard, Danielle Trowell, Shouxin Jiang, Weichun Zeng, Shiqin Chen, Qingshan Zhang, Fei Li

**Affiliations:** 1 School of Athletic Performance, Shanghai University of Sport, Shanghai, China; 2 Department of Nutrition and Movement Sciences, NUTRIM School of Nutrition and Translational Research in Metabolism, Maastricht University Medical Centre+, Maastricht, the Netherlands; 3 School of Human Sciences (Exercise and Sport Science), The University of Western Australia, Perth, WA, Australia; 4 Centre for Sport Research, Institute for Physical Activity and Nutrition, Deakin University, Burwood, VIC, Australia; 5 Université de Lyon, UCBL1, Laboratoire Interuniversitaire de Biologie de la Motricité - EA 7424, UFRSTAPS, 27-29 boulevard du 11 novembre 1918, 69622 Villeurbanne, Cedex, France

**Keywords:** Physiology, Sport Medicine, Biomechanics

## Abstract

This study examined the relationship between lower-limb strength and cumulative neuromuscular activation (CNA) during running at different speeds in recreational runners. Twenty-three participants completed three sessions: (1) running at 10, 12, and 14 km h^−1^ while integrated electromyography (EMG) of eight lower-limb muscles was collected; (2) isokinetic peak torque (PT) assessment of the knee and ankle joints at 60°·s^−1^; and (3) isometric mid-thigh pull (IMTP) evaluation of force-time characteristics. Higher eccentric knee flexor PT correlated with lower CNA of the biceps femoris at 12 km h^−1^ (r = −0.60, *p* < 0.05) and 14 km h^−1^ (r = −0.64, *p* < 0.05), while higher IMTP rate of force development (RFD) was associated with lower gastrocnemius lateralis CNA at 14 km h^−1^ (r = −0.58 to −0.63, *p* < 0.05). These results suggest that greater eccentric knee flexor strength and rapid force production may enhance neuromuscular efficiency by reducing cumulative activation demands during running.

## Highlights

• Greater eccentric knee flexor strength reduced biceps femoris activation
• Higher isometric RFD reduced gastrocnemius activation at high speed
• Strength-related reductions in activation increased with speed

## Graphical abstract

**Figure:**
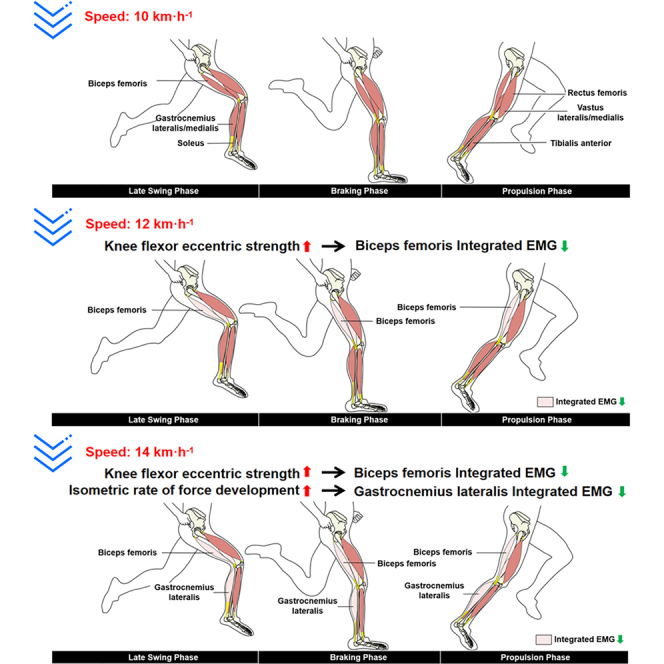

## Introduction

During running, neuromuscular activation results from the coordinated, cyclic activation of multiple lower-limb muscles that together absorb impact, generate propulsive force, and maintain postural stability.^1^^,^^2^ As a result, neuromuscular activation plays a crucial role in determining both energy expenditure and muscle fatigue.^3^^,^^4^^,^^5^ Lower-limb neuromuscular activation patterns during running are influenced by various biomechanical and contextual factors, such as running speed, stride frequency, posture, footwear, running surface, fatigue level, and training background.^6^^,^^7^^,^^8^ Biomechanically, studies have shown that both running speed and stride frequency significantly affect muscle activation, altering the amplitude and the timing of activation onset and offset.^9^^,^^10^ Surface electromyography (EMG) studies of lower-limb muscles consistently show that as the running speed increases, the amplitude of activation increases, and activation bursts occur earlier in the gait cycle.^10^ Stride frequency is another key factor influencing muscle activation, with higher frequencies shifting activation toward the late swing phase.^9^ However, most studies to date have determined the peak, or the average muscle activation during the ground contact phase only, or during a gait cycle, without considering the influence of stride frequency.^11^ While some studies have found correlations between the magnitude and timing of neuromuscular activation and the energy cost of prolonged running, their results are inconsistent.^4^^,^^11^ These inconsistencies likely stem, at least in part, from methodological differences in quantifying neuromuscular activation. Existing methods do not account for variations in the duration of the ground contact phase or gait cycle (e.g., due to differences in stride frequency), which could reduce their sensitivity in linking neuromuscular activation to running economy or fatigue. For example, although neuromuscular activation may be lower per gait cycle with a higher stride frequency, the overall neuromuscular activation required to run a given time or distance may be higher if a greater number of gait cycles is used. To account for such effects, the surface electromyographic signal can be integrated over specific time or distance intervals to enable comparison on a unified temporal scale (e.g., per minute) rather than per gait cycle.^12^^,^^13^ The resulting iEMG (integrated EMG), derived from temporal integration of the raw EMG signal, reflects the cumulative neuromuscular activation (CNA), typically measured in μV·s.^13^^,^^14^ This approach may not only improve the sensitivity of assessing the link between neuromuscular activation, running economy, and fatigue^13^^,^^15^^,^^16^ but also provides a time-standardized framework applicable to sports performance analysis, clinical gait assessment, and wearable monitoring technologies.^14^^,^^17^^,^^18^

Different abilities—such as strength—may influence the muscle activation pattern during running. In particular, muscle strength appears to be a key determinant of the neuromuscular activation required to produce a given force, as this relationship is strongly influenced by muscle hypertrophy and neuromuscular efficiency.^19^ Specifically, stronger muscles (i.e., with myofibrillar hypertrophy) require recruitment of fewer fibers to generate the same force, thus reducing neuromuscular activation.^19^ Further, improvements in neuromuscular efficiency and reductions in antagonist co-activation, often seen in strength-trained individuals, can further lower activation of the agonist muscles.^20^^,^^21^ Research combining muscle strength assessments with measurements of neuromuscular activation (such as EMG during dynamometer use) supports this view. For instance, Balshaw and colleagues observed that long-term (4 years) resistance-trained individuals produced a given knee extension moment with lower neuromuscular activation as compared to untrained individuals.^21^ Similar findings were reported in another study in the elbow flexors, where resistance-trained individuals achieved a given increase in force with a smaller increase in neuromuscular activation.^22^ However, although such findings have highlighted the impact of muscle strength on neuromuscular activation during brief, isolated contractions, it is still unclear whether these effects persist during prolonged, repetitive contractions typical of endurance activities like running. A similar effect may, however, be expected during running for the following reason. Strength training can lead to fiber hypertrophy, including in type I fibers,^23^ which are primarily recruited during submaximal running. Type I fiber hypertrophy may reduce the need to recruit higher-threshold motor units, which are less efficient and more prone to fatigue.^23^^,^^24^ Furthermore, an increased rate of force development (RFD) may reflect an enhanced neuromuscular efficiency, resulting from the central nervous system’s improved ability to optimally activate motoneurons and muscle fibers.^25^ This allows fewer motor units to be recruited at a given running speed.^25^ However, no studies have yet directly examined the relationship between lower-limb strength, rapid force production, and CNA during prolonged running.

Therefore, the aim of this study is to examine the relationships between knee and ankle joint peak torque (PT) and rapid force production and iEMG of eight lower-limb muscles across multiple running speeds in recreational male runners. We investigated these relationships for the knee and ankle joint due to their important roles in supporting and accelerating body mass during running.^26^ Furthermore, because evidence suggests that muscle strength becomes increasingly important at higher running speeds,^27^ we also investigated the relationship between muscle strength and CNA across varying running speeds. We hypothesized that runners exhibiting greater lower-limb strength and rapid force production would exhibit lower CNA at each running speed, with the relationship becoming stronger at higher running speeds (e.g., >12 km h^−1^) due to increased neuromuscular demands. Confirming this relationship could advance training prescription in runners, guide rehabilitation strategies, and support the development of real-time neuromuscular monitoring tools.

## Results

After applying the Benjamini-Hochberg correction for multiple comparisons, several correlations remained statistically significant between knee joint isokinetic strength, ankle joint isokinetic strength, isometric mid-thigh pull (IMTP) force-time characteristics, and lower-limb CNA.

### Isokinetic strength and CNA

**Figure 1:**
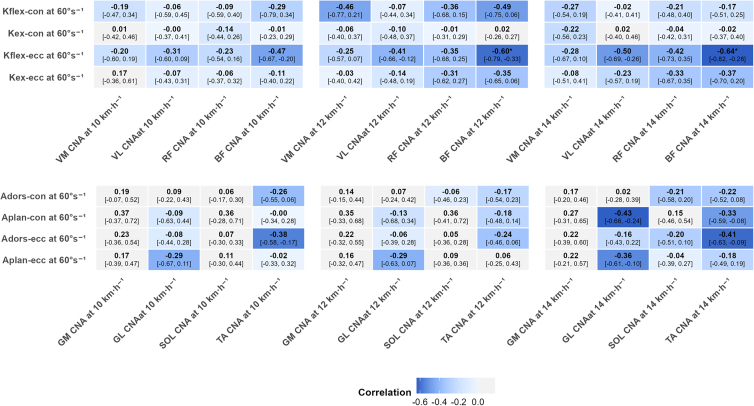
Correlation coefficients between lower-limb joint strength at 60°·s^−1^ and muscle CNA at 10, 12, and 14 km h^−1^ Heatmap displaying the relationships at 10, 12, and 14 km h^−1^. Values in brackets represent 95% bootstrap confidence intervals. Data were analyzed using Pearson correlation coefficients (*n* = 23 participants). Asterisk indicates statistical significance (*p* < 0.05). K_flex-con_, knee flexor muscle relative peak torque in concentric action; K_ex-con_, knee extensor muscle relative peak torque in concentric action; K_flex-ecc_, knee flexor muscle relative peak torque in eccentric action; K_ex-ecc_, knee extensor muscle relative peak torque in eccentric action; A_dors-con_, dorsiflexor muscle relative peak torque in concentric action; A_plan-con_, plantar flexor muscle relative peak torque in concentric action; A_dors-ecc_, dorsiflexor muscle relative peak torque in eccentric action; A_plan-ecc_, plantar flexor muscle relative peak torque in eccentric action.

**Figure 2:**
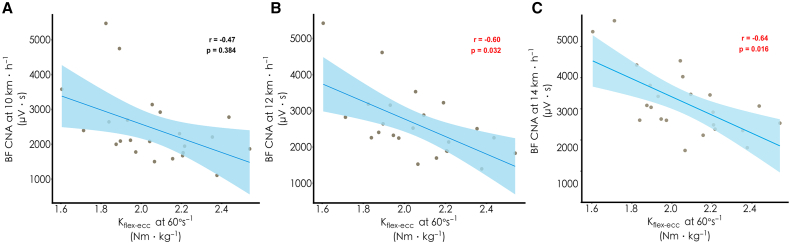
Correlations between K_flex-ecc_ and BF CNA at varying running speeds Scatterplots displaying the relationship at (A) 10 km h^−1^, (B) 12 km h^−1^, and (C) 14 km h^−1^. Red font indicates statistical significance (*p* < 0.05) determined by Pearson correlation test. Data points represent individual participants (*n* = 23).

As shown in Figure 1, for knee joint isokinetic strength, knee flexor eccentric (K_flex-ecc_) PT showed a large negative correlation with biceps femoris (BF) CNA at 12 km h^−1^ (r = −0.60, *p* = 0.032) and 14 km h^−1^ (r = −0.64, *p* = 0.016), with a directionally similar but non-significant relationship at 10 km h^−1^ (r = −0.47, *p* = 0.384) (Figure 2). No significant correlations were found between other knee strength parameters—knee flexor concentric (K_flex-con_) and knee extensor concentric (K_ex-con_) and eccentric (K_ex-ecc_) PT—and CNA—vastus lateralis (VL), vastus medialis (VM), and rectus femoris (RF)—at 10 km h^−1^ (r = −0.31 to 0.18, *p* ≥ 0.971), 12 km h^−1^ (r = −0.49 to −0.02, *p* ≥ 0.139), and 14 km h^−1^ (r = −0.42 to 0.03, *p* ≥ 0.376), although most effect directions were negative. For ankle joint isokinetic strength, no significant correlations were found between ankle PT measurements—ankle dorsiflexor concentric (A_dors-con_) and eccentric (A_dors-ecc_) and plantar flexor concentric (A_plan-con_) and eccentric (A_plan-ecc_) PT—and CNA—gastrocnemius medialis (GM), gastrocnemius lateralis (GL), soleus (SOL), and tibialis anterior (TA)—at 10 km h^−1^ (r = −0.38 to 0.37, *p* ≥ 0.469), 12 km h^−1^ (r = −0.29 to 0.36, *p* ≥ 0.824), and 14 km h^−1^ (r = −0.43 to 0.27, *p* ≥ 0.400).

### Isometric force-time characteristics and CNA

**Figure 3:**
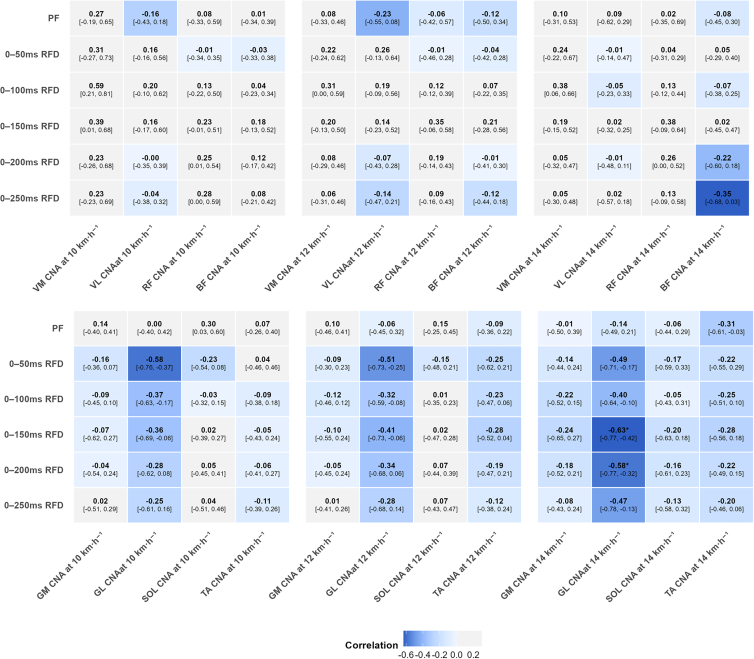
Correlation coefficients between isometric force-time characteristics and muscle CNA at 10, 12, and 14 km h^−1^ Heatmap displaying the relationships at 10, 12, and 14 km h^−1^. Values in brackets represent 95% bootstrap confidence intervals. Data were analyzed using Pearson correlation coefficients (*n* = 23 participants). Asterisk indicates statistical significance (*p* < 0.05). PF, relative peak force.

**Figure 4:**
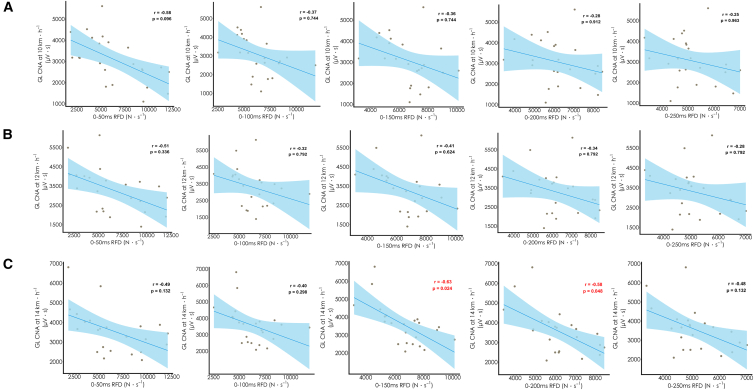
Correlations between IMTP 0–50 ms to 0–250 ms RFD and GL CNA at varying running speeds Scatterplots displaying the relationship at (A) 10 km h^−1^, (B) 12 km h^−1^, and (C) 14 km h^−1^. Red font indicates statistical significance (*p* < 0.05) determined by Pearson correlation test. Data points represent individual participants (*n* = 23).

As shown in Figure 3, the IMTP force-time characteristics, relative peak force (PF), and RFD across all time intervals showed no significant correlation with knee joint CNA at any of the three speeds (r = −0.35 to 0.59, *p* ≥ 0.072). Regarding ankle CNA, RFD from 0–150 ms to 0–200 ms showed large negative correlations with GL CNA at 14 km h^−1^ (r = −0.58 to −0.63, *p* = 0.024–0.048). However, no significant correlations were observed at 10 km h^−1^ (r = −0.28 to −0.36, *p* = 0.744–0.912) or 12 km h^−1^ (r = −0.34 to −0.41, *p* = 0.624–0.792) (Figure 4). Similarly, PF and RFD across all time intervals showed no significant correlation with other ankle muscles’ CNA (GM, SOL, and TA) at 10 km h^−1^ (r = −0.24 to 0.30, *p* ≥ 0.912), 12 km h^−1^ (r = −0.28 to 0.15, *p* ≥ 0.792), or 14 km h^−1^ (r = −0.31 to −0.01, *p* ≥ 0.588), although most effect directions were negative.

**Figure 5:**
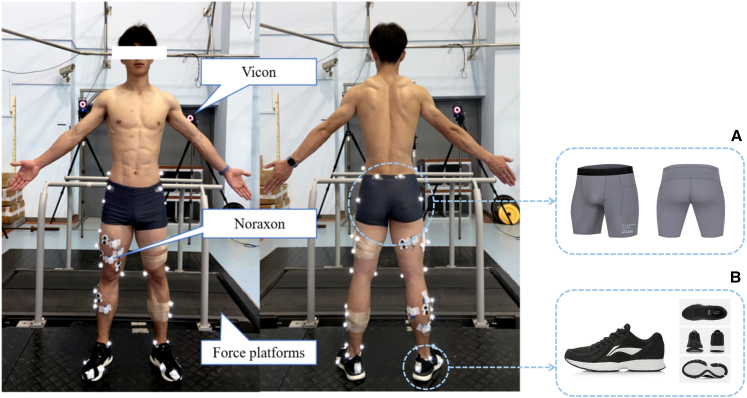
Running biomechanics and surface electromyography test Representative images showing (A) the standardized compression tights and (B) the running shoes used in the protocol.

**Figure 6:**
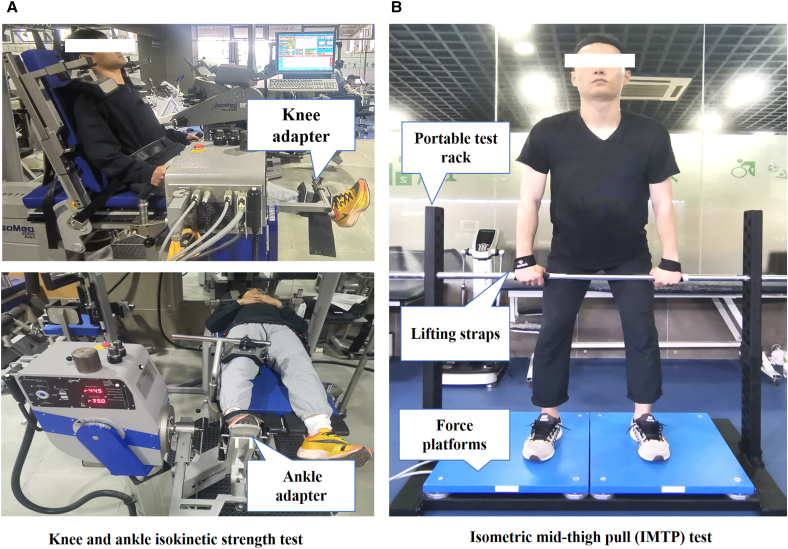
Lower-limb strength test Experimental setup for (A) the knee and ankle isokinetic strength test and (B) the IMTP test.

### Sensitivity analysis using iEMG per gait cycle

To assess whether stride frequency influenced the relationships between CNA and strength outcomes, a sensitivity analysis was conducted using iEMG per gait cycle instead of CNA. The resulting correlations with strength outcomes showed the same directional trends and comparable magnitudes to the main analyses (Tables S1–S4 in supplemental information). Specifically, greater K_flex-ecc_ PT was correlated with lower BF iEMG per gait cycle at 12 km h^−1^ (r = −0.61, *p* < 0.05) and 14 km h^−1^ (r = −0.65, *p* < 0.001), and higher RFD during the IMTP in the 0- to 150-ms (r = −0.64, *p* < 0.05) and 0- to 200-ms (r = −0.56, *p* = 0.058) intervals was significantly correlated with lower GL iEMG per gait cycle at 14 km h^−1^.

## Discussion

This study investigated the relationship between lower-limb strength parameters, including isokinetic strength and isometric force-time characteristics, and CNA in recreationally trained male runners (Figures 5 and 6). The primary findings indicate that higher knee flexor eccentric PT is correlated with lower BF CNA at 12 and 14 km h^−1^ (|r| ≥ 0.55), with a similar but non-significant trend observed at 10 km h^−1^. Likewise, higher RFD during the IMTP in the 0- to 150-ms and 0- to 200-ms intervals is significantly correlated with lower CNA of the GL at 14 km h^−1^ (|r| ≥ 0.55), with a similar non-significant trend at 10 and 12 km h^−1^. While correlations between other strength indicators and the CNA of most lower-limb muscles were not significant, they generally also indicated greater strength being associated with lower CNA. Importantly, a sensitivity analysis using iEMG per gait cycle produced results consistent with those obtained using CNA, suggesting that the observed associations were driven by the muscle activation patterns per gait cycle itself rather than by stride frequency. These findings collectively suggest a prominent influence of knee flexor eccentric strength and lower-limb rapid force production on CNA, in particular at higher running speeds, with a smaller (but possibly still meaningful) influence of strength and rapid force production on CNA for other muscles at the investigated speeds.

### Maximal force and cumulative neuromuscular activation

The present study found that maximal knee flexor eccentric strength was inversely associated with BF CNA at running speeds of 12 km h^−1^ (r = −0.60) and 14 km h^−1^ (r = −0.64), with a similar directional, but non-significant, relationship at 10 km h^−1^ (r = −0.47). Although the correlations for most other knee muscles investigated were directionally similar, the statistical power to detect these associations was limited with the current sample size. Nevertheless, these observations largely support our hypothesis that stronger muscles require lower CNA during running, in line with previous observations during resistance training exercises.^21^^,^^22^ Mechanistically, strength training induces hypertrophy of muscle fibers, including the type I fibers predominantly recruited during submaximal running intensities.^23^ This hypertrophy enhances the fibers’ absolute force-generating capacity, thereby decreasing the need to recruit higher-threshold motor units during running.^24^

The reason why a relationship between greater maximal strength and lower CNA was most prominently observed for the knee flexors, as opposed to other muscles, may be due to their primary role in propulsion and decelerating the lower limb, rather than in supporting body mass.^15^^,^^26^ Specifically, the *hamstring* muscles brake knee extension during the swing phase before foot strike.^4^^,^^28^ In contrast, muscles such as the knee extensors and plantar flexors are primarily responsible for supporting body mass.^4^^,^^26^ While increases in fiber cross-sectional area (and thereby strength) for these latter muscles may on the one hand reduce CNA, the increased overall body mass may increase CNA requirements, thereby offsetting part of the lower activation due to greater strength, and in turn reducing the strength of correlations between maximal muscle strength and CNA for these muscles. The reason why knee flexor eccentric strength as opposed to concentric strength better correlated with CNA may relate to the higher similarity of an eccentric contraction type during the isokinetic testing with the possible eccentric contraction type during the late swing phase.^28^^,^^29^ In contrast to the significant relationship found for the knee flexors, no significant correlations were observed between isokinetic knee extensor strength and the CNA of the quadriceps (RF, VL, and VM), or between isokinetic ankle joint strength and the CNA of *triceps surae* (GM, GL, and SOL) or TA. This lack of correlation may at least partly be due to differences in contraction patterns: during running, the *quadriceps* and *triceps surae* predominantly contract (quasi-) isometrically,^30^ whereas isokinetic tests typically measure concentric or eccentric actions. (Quasi-) isometric and dynamic muscle actions have neural and mechanical differences that may not allow an individual to fully express their maximum force in both movements, thus reducing the strength of correlations between isokinetic strength assessments and CNA during running for the *quadriceps* and *triceps surae*.^31^ Additionally, isokinetic testing typically involves open-chain movements, while running involves a combination of closed-chain and open-chain actions.^32^ Stensdotter et al.^33^ found that quadriceps activation patterns differ between open- and closed-chain tasks. For example, during closed-chain knee extension, the quadriceps activate almost simultaneously, while in open-chain extension, the RF activates first and the VM last. Such discrepancies may also explain why isokinetic tests did not significantly correlate with CNA during dynamic running, with the exception of the BF. Nevertheless, it should be noted that also for the quadriceps, higher levels of strength were non-significantly associated with lower CNA.

Notably, our findings show that the magnitude of the correlation between maximal muscle strength and CNA increases with higher running speeds for most of the muscles investigated (compared to slower speeds, e.g., 10 km h^−1^). The reason for this may relate to the larger muscle force and activation requirements with increases in speed, thereby increasing signal-to-noise ratio, and thereby improving our ability to detect correlations.

### IMTP outcomes and calf muscle cumulative neuromuscular activation

In partial support of our hypothesis, higher IMTP RFD from 0 to 150 ms and 0 to 200 ms showed large correlations with lower GL CNA at 14 km h^−1^. Although no significant correlations were observed in other calf muscles, the direction of the effect was generally consistent across the other calf muscles and different speeds. During running, calf muscles endure forces up to 12 times the body weight as they brake and support the body’s center of mass, serving as the primary force generators for propulsion.^30^^,^^34^ As a result, these muscles are highly activated during the contact phase. Typically, RFD within 250 ms is recognized as a marker of rapid force production ability,^35^ which corresponds closely to the ground contact times observed in this study (e.g., ≤250 ms at 10–14 km h^−1^). A higher RFD could be associated with greater neuromuscular efficiency, possibly reflecting (1) enhanced neural drive for faster force production^25^ and (2) reduced co-activation of antagonists (e.g., TA), thereby decreasing the activation demand on agonists (e.g., SOL, GM, and GL).^20^ These mechanisms might partly help to interpret the association between RFD and calf CNA, although this remains speculative.

As running speed increases, the plantar flexors (i.e., SOL, GM, and GL) must generate greater force during shorter ground contact times, necessitating faster and stronger contractions.^36^ These stronger contractions may improve the ability to detect relationships between CNA and RFD. Notably, at 14 km h^−1^ (with a contact time of about 200 ms), GL exhibited stronger correlations between its CNA and RFD within the 0- to 200-ms window compared to the other plantar flexors. One possible explanation is that GL exhibits a “natural” inhibitory tendency during running (avoiding excessive activation),^37^ and individuals with higher RFD might display activation patterns consistent with this inhibition, resulting in lower GL CNA. Using sural nerve electrical stimulation, Hauglustaine et al.^37^ showed that GL’s medium-latency reflex responses (P2) are predominantly inhibitory, reducing its neuromuscular activation. This inhibition may be related to task-dependent reflex modulation, which could serve to limit excessive GL involvement in dynamic activities (e.g., running). In contrast, GM consistently shows facilitatory responses, particularly in running or hopping, with prominent P2 responses reflecting selective activation. This is possibly due to its role in generating greater force and coordination during fast movements.^37^ Thus, the observed pattern could suggest that more efficient neuromuscular control at higher speeds may coincide with greater GM recruitment and relatively lower GL activation to meet rapid force demands. Tam et al.^38^ found that increased GL CNA during the support phase correlates with poorer running economy, leading us to hypothesize that a higher RFD capacity at faster speeds might be associated with better running economy, potentially through reduced GL CNA.

In terms of IMTP PF, no significant correlation was observed with calf CNA across the tested speeds (10–14 km h^−1^). This lack of significance may stem from differences in the nature of the tests. First, IMTP PF reflects sustained force production over a longer time frame (400–600 ms),^39^ whereas running at these speeds requires rapid force generation within short ground contact times (≤250 ms), potentially reducing its ability to predict CNA. Second, as a multi-joint test involving coordinated force production at the ankle, knee, and hip, the contribution of the ankle (i.e., plantar flexors) to overall torque generation in the IMTP is comparatively limited.^39^ This further weakens the correlation between IMTP metrics (PF and RFD) and neuromuscular activation of specific muscle groups such as the plantar flexors, especially at lower running speeds. Future research might benefit from using closed-chain, isolated isometric plantar flexion tests to more directly and specifically evaluate the relationship between plantar flexor neuromuscular activation and strength production capacity, potentially improving the strength of these observed correlations.

### IMTP outcomes and knee muscle cumulative neuromuscular activation

No significant correlations were observed between IMTP indices, including RFD and PF, and the CNA of knee joint muscle groups, such as the VM, VL, RF, and BF, and the direction of the effect was inconsistent, suggesting that these strength parameters have a limited influence on knee muscle CNA during running. The lack of a significant correlation between RFD and knee muscle CNA in running may be attributed to differences in muscle function and the complexity of activation patterns. In the early phase of the stance, knee muscles (such as the quadriceps and hamstrings) are co-activated to absorb impact and stabilize the knee joint, contributing most to braking (i.e., decelerating the center of mass) and support.^26^^,^^40^ In the latter part of the stance phase, the ankle plantar flexors (such as the soleus and gastrocnemius) contribute mostly to propulsion and support.^26^ Consequently, greater RFD may reduce ankle muscle CNA through improved neuromuscular efficiency, but its effect on knee muscles is less pronounced due to their stabilizing role. This contrasts with our results regarding the significant correlation between the eccentric maximal force of the knee flexors and BF CNA during running. This may be related to the eccentric contraction of BF to decelerate the calf during the swing phase, rather than the stabilizing contribution of the knee during the support phase. Furthermore, knee muscle CNA is complex and influenced by gait characteristics such as stride frequency and stride length. Recreational runners may have greater variability in activation compared to trained runners.^41^ This increased variability could blur any direct relationship with RFD, in contrast to the more direct propulsion-driven response of the ankle plantar flexors. Similarly, IMTP PF showed no consistent relationship with knee muscle CNA across all speeds. This aligns with its limited relationship with calf muscle CNA (IMTP outcomes and calf muscle cumulative neuromuscular activation), as IMTP’s multi-joint force coordination lacks specificity for isolated knee muscle assessment and differs from the eccentric BF contraction during the swing phase (Maximal force and cumulative neuromuscular activation).

Overall, this study suggests that eccentric knee flexor isokinetic strength and the ability to rapidly generate lower-limb force are associated with reduced CNA during running, particularly for the BF at 12 and 14 km h^−1^ and the GL at 14 km h^−1^. Notably, the effects of greater muscle strength on lowering CNA during running increase with running speed. These findings suggest that improvements in lower-limb strength and rapid force production may reduce neuromuscular activation during running and potentially delay fatigue. For elite athletes, such improvements may enhance performance in high-intensity events, while for recreational runners, these gains may help manage fatigue and improve endurance during longer runs. These insights could inform the development of targeted strength training, rehabilitation programs, and wearable technologies designed to optimize performance and minimize injury risk.

### Limitations of the study

The conclusions of this study are supported by several complementary analytical approaches. In addition to examining iEMG per gait cycle, we performed a dedicated sensitivity analysis that confirmed our findings were robust regardless of whether strength was normalized to body mass or fat-free mass (see Tables S5–S8 in supplemental information). However, several limitations should be acknowledged. First, the exploratory nature of the study, coupled with the relatively small sample size, underscores the need for larger cohorts to validate these findings. Future research should also recruit female and competitive athletes and examine higher running speeds (e.g., approximately 20 km h^−1^) to yield insights more relevant to better-trained long-distance runners. Second, isokinetic joint strength was assessed at 60°·s^−1^. While this low-velocity, open-chain modality limits ecological validity with respect to running mechanics, it was specifically chosen to evaluate the maximal physiological force capacity (strength reserve) of the musculature, distinct from the rapid force capacity assessed via IMTP. However, we acknowledge that this does not fully replicate the closed-chain, dynamic nature of the gait cycle. Consequently, future studies should incorporate higher-velocity isokinetic assessments or closed-chain isometric plantar-flexion tests to better capture the functional strength demands of running. Third, although the absolute iEMG values (μV·s) were not normalized, using absolute iEMG helps preserve physiologically meaningful variations in muscle activation amplitude.^42^ Given the high homogeneity of the sample (male recreational runners aged 19–22 years), this approach was justifiable for the present exploratory analysis. Nonetheless, future studies involving larger and more heterogeneous populations should consider normalization procedures to enhance comparability. Moreover, while the present study specifically examined the relationship between force production and the CNA of individual muscles, neuromuscular co-contractions may also play a significant role in performance. Future research should, therefore, explore the potential associations between force production and co-activation during running to provide deeper insight into neuromuscular coordination mechanisms. Finally, the integrated muscle activation used in this study was employed to quantify CNA; however, it does not reflect activation during specific phases of movement. Therefore, interpretations regarding the underlying mechanisms of the observed findings should be made with caution.

## Resource availability

### Lead contact

Further information and requests for resources and reagents should be directed to and will be fulfilled by the lead contact, Fei Li (lifei@sus.edu.cn).

### Materials availability

This study did not generate new unique reagents.

### Data and code availability

• The dataset supporting the findings of this study is provided as Data S1.
• This study did not generate original code.
• No additional supporting items were generated.

## Acknowledgments

The authors would like to thank all runners who participated in this study. The study was supported by the National Social Science Fund of China (Grant No. 23CTY019), the Key Laboratory of Human Performance at Shanghai University of Sport (Grant No. 11DZ2261100), and the Research and Innovation Grant for Graduate Students, 10.13039/501100002397Shanghai University of Sport (Project No. YJSCX-2025-019). The funding sources had no involvement in the study design, data collection, data processing, manuscript writing or editing, and the decision to submit this manuscript. The results of the study are presented clearly and honestly, with no fabrication, falsification, or inappropriate data manipulation.

## Author contributions

Conceptualization, F.L. and Qingshan Zhang; methodology, F.L., Qin Zhang, and B.V.H.; investigation, Qin Zhang, S.J., W.Z., and S.C.; writing – original draft, Q.Z.; writing – review & editing, B.V.H., O.G., and D.T.; funding acquisition, F.L.; supervision, F.L. and Qingshan Zhang. All authors have read and approved the final version of the manuscript.

## Declaration of interests

The authors declare no competing interests.

## STAR★Methods

### Key resources table

**Table:** 

REAGENT or RESOURCE	SOURCE	IDENTIFIER
**Deposited data**		

Anonymized/analyzed dataset	This paper	Data S1

**Software and algorithms**		

SPSS Statistics Version 27.0	IBM	Version 27.0; RRID:SCR_002865; https://www.ibm.com/products/spss-statistics
MATLAB R2023a	MathWorks	R2023a; RRID:SCR_001622; https://www.mathworks.com/products/matlab.html
G∗Power Version 3.1.9.7	Heinrich-Heine-Universität Düsseldorf	Version 3.1.9.7; RRID:SCR_013726; https://www.psychologie.hhu.de/

**Other**		

Force plates	Kistler	Cat#9287B
Wireless surface EMG system	Noraxon	N/A
Isokinetic dynamometer	D&R Ferstl	N/A

### Experimental model and study participant details

#### Participants

A total of 29 male runners were initially recruited from a university endurance club. Six participants withdrew, resulting in a final sample of 23 runners. The participants’ physical characteristics were as follows (mean ± SD): age 20.9 ± 1.3 years, height 1.80 ± 0.06 m, and body mass 72.3 ± 10.3 kg. All participants were of Han Chinese ancestry and self-identified as Asian (race) and Chinese (ethnicity). A sensitivity analysis for the planned correlation tests was performed using G∗Power software (version 3.1.9.7). Based on the final sample size (*n* = 23), an α level of 0.05 (two-tailed), and a desired statistical power of 0.80, the analysis indicated that correlation coefficients of |r| ≥ 0.55 could be reliably detected.^43^

Inclusion criteria were: (1) at least two years of experience in endurance events; (2) participation in collegiate-level competitions ranging from 5 to 21 km; (3) over 12 months of systematic training; and (4) consistent weekly training volume of 20–30 km for the past 3 months. All participants completed health screening via the Physical Activity Readiness Questionnaire (PAR-Q) to confirm their eligibility. The experimental protocol received approval from the Ethics Committee of Shanghai University of Sport, China (ID: 102772023RT107). The experiments were conducted in accordance with the ethical standards of the Declaration of Helsinki. All participants provided informed consent after being fully briefed on the procedures and potential risks.

### Method details

#### Study design

This study employed an exploratory cross-sectional design to investigate potential associations between CNA during running and lower-limb strength characteristics in recreational runners. Participants completed three laboratory-based assessments. First, anthropometric measurements were taken, followed by the measurement of CNA during treadmill running at speeds of 10, 12, and 14 km h^−1^. Next, isokinetic strength testing (60°·s^−1^) assessed peak torque during concentric and eccentric contractions of the knee and ankle muscles. The protocol concluded with an IMTP, which was used to measure force-time characteristics including PF and RFD at distinct time intervals (0–50 ms to 0–250 ms). The RFD during the IMTP was measured because it reflects the rapid force production capacity of the lower limbs during the brief ground contact phase of running, a factor linked to running performance. Participants followed a standardized pre-testing protocol, which included 48 h of exercise restriction with at least 8 h of sleep per day, as verified through questionnaire screening.

#### Measurement procedures

##### Running biomechanics and surface electromyography test

As shown in Figure 5, participants were tested in standardized running shoes and compression tights. Two Kistler force plates (model 9287 B, 90 × 60 × 10 cm) were installed under the treadmill to collect vertical ground reaction forces (vGRF) at 1,000 Hz. CNA from eight right-lower-limb muscles was recorded using the Noraxon Ultium wireless surface electromyography system (2,000 Hz sampling frequency, 24-bit ADC, 20 mm inter-electrode distance) for the following muscles: VL, VM, RF, BF, GM, GL, SOL, and TA muscles. After skin preparation and electrode placement according to SENIAM guidelines (http://www.seniam.org),^44^ including shaving, abrasion, and cleaning with alcohol to ensure low impedance and reproducible signal quality, participants performed a 4-min warm-up at 8 km h^−1^, followed by 4-min running bouts at 10 km h^−1^ (2.78 m s^−1^), 12 km h^−1^ (3.33 m s^−1^), and 14 km h^−1^ (3.89 m s^−1^), with 1-min intervals between speeds.^45^ Running speed increased in a fixed order to ensure a gradual and controlled progression of workload, consistent with previous running economy testing protocols.^46^ This approach was chosen to avoid abrupt speed transitions that might disrupt gait stability. Each 4-min stage allowed participants to reach stable physiological and biomechanical conditions,^47^^,^^48^ while the 1-min intervals provided brief recovery to avoid fatigue.^48^ Data were collected for 15 s during the final minute of each speed, synchronously recorded via an analog-to-digital converter. The vGRF raw data were filtered using a second-order bidirectional Butterworth low-pass filter with a cutoff frequency of 50 Hz. A vertical force signal of 50 N was used to determine the beginning and end of the ground contact phase, which is commonly used in gait analysis.^15^ The raw EMG signal was processed in MATLAB R2023a using a Butterworth band-pass filter (20–400 Hz) to remove motion artifacts, followed by full-wave rectification to obtain absolute values.^49^ A standardized temporal method was then applied to calculate the CNA of running, rather than per individual gait cycle. The mean iEMG per gait cycle was calculated from 10 representative gait cycles.^49^ Prior to calculation, a visual inspection was performed to strictly exclude strides exhibiting obvious motion artifacts, sudden baseline shifts, or irregular gait patterns (e.g., stumbling). This ensured that the analyzed data accurately reflected the stable neuromuscular state. The iEMG value was then multiplied by the stride frequency (strides·min^−1^) to normalise the outcome and obtain the CNA, as shown in Equation 1. This adjustment accounts for stride frequency variations across running speeds, enabling direct quantification of CNA during prolonged running on a unified temporal scale (per minute) rather than on a per gait cycle basis.

(Equation 1) CNA=iEMGpergaitcycle×Stridefrequency

$$iEMG=\sum_{i=1}^{N}\left|x_{i}\right|$$

$$\text{Stride}\;\text{frequency}=\frac{60}{T_{c}+T_{f}}$$

where T_c_ is the contact time (s), and T_f_ is the flight time (s).

#### Isokinetic strength test

Knee and ankle muscle PT was assessed using a calibrated dynamometer (IsoMed 2000, D&R Ferstl GmbH, Hemau, Germany) (Figure 6A). The instrument was calibrated before each test according to the manufacturer’s instructions. The dynamometer shaft was positioned horizontally for automatic gravity compensation, with the operator avoiding manual contact during weighing. Participants remained relaxed to eliminate gravity effects. After three submaximal familiarization trials, participants performed five concentric and eccentric contractions at 60° s^−1^, a standard speed for reliable assessment of maximal muscle force capacity.^32^^,^^50^ The highest PT recorded was used in the subsequent analyses. Rest periods of 1 min between contraction types and 10 min between joints were provided. Verbal encouragement was provided to ensure maximal effort. Relative peak torque was calculated as maximum PT divided by body mass.

For knee joint torque assessment, participants sat with approximately 85 °hip flexion, with the backrest adjusted for optimal knee flexion/extension. The right knee’s range of motion was set from full extension to 90° flexion, with participants gripping handles for stability.^51^ K_flex-con_, K_flex-ecc_, and K_ex-con_, K_ex-ecc_ PT were measured.

For ankle testing, participants lay supine with fully extended knees and hips. The right foot was secured to the ankle adapter, with thighs, hips, and shoulders fixed. The dynamometer shaft was aligned with the lateral malleolus, with the ankle in a neutral position (0°) and a range from 15° dorsiflexion to 40° plantar flexion.^52^ A_dors-con_, A_dors-ecc_, and A_plan-con_, A_plan-ecc_ PT were measured.

#### Isometric mid-thigh pull test

The IMTP test allows measurement of PF and RFD in time intervals relevant to ground contact, and has been validated as a method to assess (explosive) lower-limb strength. The IMTP was assessed using dual force platforms (9290AA; Kistler, Winterthur, Switzerland, 1,000 Hz) and a portable rack (Figure 6B), which previously have been shown to exhibit high reliability (ICC >0.95).^35^ Participants set the crossbar at the clean second pull position (hip flexion 140–150°, knee flexion 125–145°), using overhand grip with lifting straps.^39^ After four progressive warm-up trials (50%, 70%, 80%, and 90% of maximum effort, 60 s intervals), participants performed two maximal IMTP tests, instructed to *“Push the ground fast and hard with maximum effort”*.^39^ Each trial consisted of 2 s of static standing (without any pre-tension; only body weight was applied, i.e., no force was applied to the bar during this period), followed by 5 s of maximal pulling with verbal encouragement. There was a 3-min rest period between trials. A threshold of 5 standard deviations above body weight was used to determine the onset for time-specific force values and RFD calculations, in order to ensure data accuracy by accounting for signal noise during the weighing period.^39^ The force-time series with the highest PF was included in the statistical analysis. Force-time curves were used to determine peak force (PF relative to body mass), and the RFD was calculated during epochs representative of ground contact times in submaximal running speeds using Equation 2:

(Equation 2) $$\text{RFD}=\frac{\Delta \text{Force}}{\Delta \text{Time}}$$

The RFD was computed during the following predetermined time windows: 0–50 to 0–250 ms (0–50 ms RFD, 0–100 ms RFD, 0–150 ms RFD, 0–200 ms RFD and 0–250 ms RFD).^35^ These time intervals were selected based on the time that the runners in this study spent on the ground at a speed of 10–14 km h^−1^.

### Quantification and statistical analysis

Data normality was assessed using the Shapiro-Wilk test, with values expressed as mean ± SD. Pearson correlation analyses were used to examine the relationships between isokinetic joint strength (concentric and eccentric PT of knee and ankle joints), IMTP force-time characteristics (PF and RFD at 0–50 to 0–250 ms), and lower-limb CNA (VM, VL, RF, BF, GM, GL, SOL, and TA). To assess whether stride frequency confounded the CNA results (calculated as iEMG per gait cycle × stride frequency), a sensitivity analysis was performed by repeating all correlation analyses using iEMG per gait cycle alone, independent of stride frequency. Correlation coefficients (r) were interpreted as *small* (0.1–0.3), *moderate* (0.3–0.5), *large* (0.5–0.7), *very large* (0.7–0.9), and *nearly perfect* (0.9–1.0).^53^ The Benjamini-Hochberg procedure was used to control the false discovery rate, with statistical significance set at *p* < 0.05. For transparency, bootstrap 95% confidence intervals were also reported. All analyses were performed using SPSS (version 27.0; IBM Corp., Armonk, NY, USA). Unless otherwise stated, *n* = 23 and represents the number of participants; exact n and all statistical details (test used, r, confidence intervals, and adjusted *p* values) are reported in the results and/or figure legends.

### Additional resources

This paper does not report any additional resources.

## Supplemental information

Document S1. Tables S1–S8

Data S1. Lower-limb strength and integrated EMG data for all participants under all experimental conditions in this study, related to Figures 1–4
