# Supplementary material for: Exploring the relationship between lower-limb strength and neuromuscular activation in running: Insights from integrated EMG
Source: iScience. 2026 Mar 11;29(4):115232. doi: 10.1016/j.isci.2026.115232 (PMC13053773; doi:10.1016/j.isci.2026.115232)
Supplement: Document S1. Tables S1–S8 [file mmc1.pdf]

iScience, Volume *29*

Supplemental information

Exploring the relationship between lower-limb strength and neuromuscular activation in running: Insights from integrated EMG

Qin Zhang, Bas Van Hooren, Olivier Girard, Danielle Trowell, Shouxin Jiang, Weichun Zeng, Shiqin Chen, Qingshan Zhang, and Fei Li

Table S1. Correlation coefficients between knee joint strength at 60°·s^{-1} and knee muscles' iEMG per gait cycle at 10, 12, and 14 km·h^{-1}. Related to Figure 1.

	VM iEMG at 10 km·h^{-1}	VL iEMG at 10 km·h^{-1}	RF iEMG at 10 km·h^{-1}	BF iEMG at 10 km·h^{-1}	VM iEMG at 12 km·h^{-1}	VL iEMG at 12 km·h^{-1}	RF iEMG at 12 km·h^{-1}	BF iEMG at 12 km·h^{-1}	VM iEMG at 14 km·h^{-1}	VL iEMG at 14 km·h^{-1}	RF iEMG at 14 km·h^{-1}	BF iEMG at 14 km·h^{-1}
$K_{flex\text{-}con}$ at 60°s^{-1} (Nm·kg^{-1})	-0.24<br>(-0.50 to 0.25)	-0.11<br>(-0.63 to 0.42)	-0.16<br>(-0.65 to 0.30)	-0.34<br>(-0.83 to 0.32)	-0.49<br>(-0.78 to 0.16)	-0.08<br>(-0.47 to 0.35)	-0.41<br>(-0.68 to 0.17)	-0.51<br>(-0.78 to 0.14)	-0.32<br>(-0.59 to 0.14)	0.03<br>(-0.34 to 0.28)	-0.25<br>(-0.51 to 0.27)	-0.21<br>(-0.57 to 0.22)
$K_{ex\text{-}con}$ at 60°s^{-1} (Nm·kg^{-1})	0.02<br>(-0.42 to 0.49)	-0.01<br>(-0.38 to 0.40)	-0.15<br>(-0.50 to 0.22)	-0.03<br>(-0.27 to 0.26)	-0.07<br>(-0.40 to 0.33)	-0.10<br>(-0.48 to 0.30)	-0.03<br>(-0.33 to 0.23)	0.01<br>(-0.25 to 0.28)	-0.24<br>(-0.60 to 0.23)	-0.29<br>(-0.52 to 0.40)	-0.05<br>(-0.44 to 0.25)	-0.05<br>(-0.42 to 0.38)
$K_{flex\text{-}ecc}$ at 60°s^{-1} (Nm·kg^{-1})	-0.22<br>(-0.57 to 0.16)	-0.31<br>(-0.60 to 0.02)	-0.25<br>(-0.54 to 0.13)	-0.47<br>(-0.67 to -0.20)	-0.27<br>(-0.56 to 0.06)	-0.41<br>(-0.68 to -0.12)	-0.38<br>(-0.67 to 0.31)	**-0.61***<br>**(-0.79 to -0.29)**	-0.31<br>(-0.66 to 0.07)	-0.21<br>(-0.67 to -0.14)	-0.44<br>(-0.76 to 0.23)	**-0.65***<br>**(-0.83 to -0.37)**
$K_{ex\text{-}ecc}$ at 60°s^{-1} (Nm·kg^{-1})	0.20<br>(-0.37 to 0.64)	-0.05<br>(-0.46 to 0.33)	-0.04<br>(-0.38 to 0.33)	-0.11<br>(-0.37 to 0.22)	-0.01<br>(-0.40 to 0.40)	-0.13<br>(-0.46 to 0.22)	-0.30<br>(-0.60 to 0.26)	-0.35<br>(-0.64 to 0.08)	-0.07<br>(-0.54 to 0.43)	-0.06<br>(-0.47 to 0.13)	-0.34<br>(-0.66 to 0.29)	-0.37<br>(-0.72 to 0.17)

Table displaying the relationships at 10, 12, and 14 km·h−1. Values in brackets represent 95% bootstrap confidence intervals. Data were analyzed using Pearson correlation coefficients (n = 23 participants). *indicates statistical significance ($p < 0.05$). Bold font indicates $|r| \geq 0.55$. Abbreviations: iEMG, integrated EMG; VM, vastus medialis; VL, vastus lateralis; RF, rectus femoris; BF, biceps femoris; Kflex-con, knee flexor muscles relative peak torque in concentric action; Kex-con, knee extensor muscles relative peak torque in concentric action; Kflex-ecc, knee flexor muscles relative peak torque in eccentric action; Kex-ecc, knee extensor muscles relative peak torque in eccentric action.

Table S2. Correlation coefficients between ankle joint strength at 60°·s^{-1} and ankle muscles' iEMG per gait cycle at 10, 12, and 14 km·h^{-1}. Related to Figure 1.

	GM iEMG at 10 km·h^{-1}	GL iEMG at 10 km·h^{-1}	SOL iEMG at 10 km·h^{-1}	TA iEMG at 10 km·h^{-1}	GM iEMG at 12 km·h^{-1}	GL iEMG at 12 km·h^{-1}	SOL iEMG at 12 km·h^{-1}	TA iEMG at 12 km·h^{-1}	GM iEMG at 14 km·h^{-1}	GL iEMG at 14 km·h^{-1}	SOL iEMG at 14 km·h^{-1}	TA iEMG at 14 km·h^{-1}
$A_{dors\text{-}con}$ at 60°s^{-1} (Nm·kg^{-1})	0.15<br>(-0.10 to 0.51)	0.06<br>(-0.30 to 0.40)	0.01<br>(-0.26 to 0.22)	-0.27<br>(-0.54 to 0.01)	0.11<br>(-0.20 to 0.42)	0.05<br>(-0.27 to 0.40)	-0.09<br>(-0.56 to 0.24)	-0.18<br>(-0.54 to 0.22)	0.15<br>(-0.24 to 0.44)	0.02<br>(-0.31 to 0.41)	-0.21<br>(-0.56 to 0.22)	-0.22<br>(-0.50 to 0.07)
$A_{plan\text{-}con}$ at 60°s^{-1} (Nm·kg^{-1})	0.39<br>(-0.33 to 0.70)	-0.05<br>(-0.62 to 0.48)	0.40<br>(-0.28 to 0.72)	0.03<br>(-0.28 to 0.32)	0.36<br>(-0.30 to 0.68)	-0.09<br>(-0.66 to 0.38)	0.38<br>(-0.39 to 0.69)	-0.15<br>(-0.47 to 0.17)	0.28<br>(-0.31 to 0.68)	-0.42<br>(-0.69 to -0.16)	0.16<br>(-0.49 to 0.58)	-0.33<br>(-0.60 to -0.04)
$A_{dors\text{-}ecc}$ at 60°s^{-1} (Nm·kg^{-1})	0.23<br>(-0.32 to 0.53)	-0.07<br>(-0.45 to 0.29)	0.07<br>(-0.31 to 0.31)	-0.36<br>(-0.57 to -0.14)	0.21<br>(-0.29 to 0.54)	-0.05<br>(-0.40 to 0.30)	0.05<br>(-0.36 to 0.29)	-0.22<br>(-0.46 to 0.09)	0.24<br>(-0.32 to 0.60)	-0.11<br>(-0.41 to 0.34)	-0.12<br>(-0.46 to 0.20)	-0.36<br>(-0.61 to -0.01)
$A_{plan\text{-}ecc}$ at 60°s^{-1} (Nm·kg^{-1})	0.19<br>(-0.32 to 0.47)	-0.25<br>(-0.65 to 0.12)	0.14<br>(-0.28 to 0.45)	0.02<br>(-0.29 to 0.33)	0.18<br>(-0.25 to 0.48)	-0.24<br>(-0.62 to 0.09)	0.11<br>(-0.35 to 0.36)	0.09<br>(-0.23 to 0.44)	0.24<br>(-0.18 to 0.55)	-0.31<br>(-0.58 to 0.01)	0.03<br>(-0.33 to 0.35)	-0.15<br>(-0.49 to 0.25)

Table displaying the relationships at 10, 12, and 14 km·h−1. Values in brackets represent 95% bootstrap confidence intervals. Data were analyzed using Pearson correlation coefficients (n = 23 participants). Abbreviations: iEMG, integrated EMG; GM, gastrocnemius medialis; GL, gastrocnemius lateralis; SOL, soleus; TA, tibialis anterior; Adors-con, dorsiflexor muscles relative peak torque in concentric action; Aplan-con, plantar flexor muscles relative peak torque in concentric action; Adors-ecc, dorsiflexor muscles relative peak torque in eccentric action; Aplan-ecc, plantar flexor muscles relative peak torque in eccentric action.

Table S3. Correlation coefficients betweenIsometric Force-Time Characteristics and knee muscles' iEMG per gait cycle at 10, 12, and 14 km·h^{-1}. Related to Figure 3.

	VM iEMG at 10 km·h^{-1}	VL iEMG at 10 km·h^{-1}	RF iEMG at 10 km·h^{-1}	BF iEMG at 10 km·h^{-1}	VM iEMG at 12 km·h^{-1}	VL iEMG at 12 km·h^{-1}	RF iEMG at 12 km·h^{-1}	BF iEMG at 12 km·h^{-1}	VM iEMG at 14 km·h^{-1}	VL iEMG at 14 km·h^{-1}	RF iEMG at 14 km·h^{-1}	BF iEMG at 14 km·h^{-1}
PF (N·kg^{-1})	0.27<br>(-0.19 to 0.63)	-0.15<br>(-0.43 to 0.20)	0.08<br>(-0.36 to 0.54)	0.01<br>(-0.34 to 0.38)	0.06<br>(-0.36 to 0.44)	-0.23<br>(-0.54 to 0.09)	-0.08<br>(-0.43 to 0.51)	-0.13<br>(-0.49 to 0.31)	0.12<br>(-0.27 to 0.54)	0.09<br>(-0.58 to 0.28)	0.02<br>(-0.37 to 0.68)	-0.07<br>(-0.44 to 0.30)
0-50ms RFD (N·s^{-1})	0.36<br>(-0.22 to 0.74)	0.19<br>(-0.16 to 0.60)	0.06<br>(-0.28 to 0.44)	0.01<br>(-0.29 to 0.46)	0.23<br>(-0.21 to 0.66)	0.26<br>(-0.13 to 0.64)	0.02<br>(-0.34 to 0.30)	-0.02<br>(-0.39 to 0.29)	0.23<br>(-0.30 to 0.64)	-0.01<br>(-0.17 to 0.49)	0.04<br>(-0.29 to 0.31)	0.05<br>(-0.27 to 0.39)
0-100ms RFD (N·s^{-1})	**0.62***<br>**(0.25 to 0.82)**	0.22<br>(-0.08 to 0.62)	0.17<br>(-0.18 to 0.54)	0.06<br>(-0.21 to 0.36)	0.29<br>(-0.02 to 0.59)	0.17<br>(-0.12 to 0.55)	0.12<br>(-0.10 to 0.38)	0.06<br>(-0.22 to 0.33)	0.36<br>(0.02 to 0.59)	-0.05<br>(-0.25 to 0.36)	0.13<br>(-0.13 to 0.40)	-0.09<br>(-0.39 to 0.26)
0-150ms RFD (N·s^{-1})	0.41<br>(0.06 to 0.68)	0.18<br>(-0.19 to 0.63)	0.27<br>(0.03 to 0.57)	0.21<br>(-0.11 to 0.55)	0.20<br>(-0.12 to 0.51)	0.14<br>(-0.23 to 0.53)	0.36<br>(-0.01 to 0.59)	0.22<br>(-0.27 to 0.56)	0.17<br>(-0.17 to 0.50)	0.02<br>(-0.36 to 0.26)	0.38<br>(-0.08 to 0.63)	0.01<br>(-0.46 to 0.55)
0-200ms RFD (N·s^{-1})	0.27<br>(-0.18 to 0.65)	0.03<br>(-0.32 to 0.42)	0.31<br>(0.08 to 0.58)	0.16<br>(-0.13 to 0.45)	0.09<br>(-0.29 to 0.45)	-0.06<br>(-0.42 to 0.29)	0.21<br>(-0.06 to 0.43)	0.02<br>(-0.41 to 0.33)	0.07<br>(-0.32 to 0.48)	-0.01<br>(-0.51 to 0.15)	0.27<br>(0.07 to 0.54)	-0.21<br>(-0.58 to 0.23)
0-250ms RFD (N·s^{-1})	0.27<br>(-0.17 to 0.67)	-0.01<br>(-0.36 to 0.36)	0.33<br>(0.08 to 0.62)	0.12<br>(-0.17 to 0.45)	0.07<br>(-0.30 to 0.46)	-0.12<br>(-0.46 to 0.22)	0.11<br>(-0.14 to 0.45)	-0.10<br>(-0.44 to 0.20)	0.06<br>(-0.30 to 0.49)	0.03<br>(-0.57 to 0.22)	0.15<br>(-0.07 to 0.63)	-0.34<br>(-0.66 to 0.07)

Table displaying the relationships at 10, 12, and 14 km·h−1. Values in brackets represent 95% bootstrap confidence intervals. Data were analyzed using Pearson correlation coefficients (n = 23 participants). *indicates statistical significance ($p < 0.05$). Bold font indicates $|r| \geq 0.55$. Abbreviations: iEMG, integrated EMG; VM, vastus medialis; VL, vastus lateralis; RF, rectus femoris; BF, biceps femoris; PF, relative peak force; RFD, rate of force development.

Table S4. Correlation coefficients betweenIsometric Force-Time Characteristics and ankle muscles' iEMG per gait cycle at 10, 12, and 14 km·h^{-1}. Related to Figure 3.

	GM iEMG at 10 km·h^{-1}	GL iEMG at 10 km·h^{-1}	SOL iEMG at 10 km·h^{-1}	TA iEMG at 10 km·h^{-1}	GM iEMG at 12 km·h^{-1}	GL iEMG at 12 km·h^{-1}	SOL iEMG at 12 km·h^{-1}	TA iEMG at 12 km·h^{-1}	GM iEMG at 14 km·h^{-1}	GL iEMG at 14 km·h^{-1}	SOL iEMG at 14 km·h^{-1}	TA iEMG at 14 km·h^{-1}
PF (N·kg^{-1})	0.13<br>(-0.44 to 0.40)	-0.01<br>(-0.46 to 0.36)	0.27<br>(-0.04 to 0.54)	0.07<br>(-0.27 to 0.36)	0.08<br>(-0.48 to 0.39)	-0.08<br>(-0.51 to 0.35)	0.12<br>(-0.29 to 0.45)	-0.11<br>(-0.39 to 0.23)	-0.01<br>(-0.49 to 0.43)	-0.15<br>(-0.50 to 0.23)	-0.05<br>(-0.42 to 0.33)	-0.31<br>(-0.62 to 0.01)
0-50ms RFD (N·s^{-1})	-0.12<br>(-0.34 to 0.21)	-0.53<br>(-0.74 to -0.30)	-0.16<br>(-0.43 to 0.19)	0.07<br>(-0.36 to 0.51)	-0.067<br>(-0.285 to 0.307)	-0.47<br>(-0.71 to -0.19)	-0.11<br>(-0.49 to 0.33)	-0.22<br>(-0.62 to 0.24)	-0.14<br>(-0.43 to 0.25)	-0.49<br>(-0.71 to -0.17)	-0.16<br>(-0.59 to 0.34)	-0.23<br>(-0.59 to 0.26)
0-100ms RFD (N·s^{-1})	-0.07<br>(-0.47 to 0.15)	-0.34<br>(-0.61 to -0.14)	0.02<br>(-0.28 to 0.21)	-0.06<br>(-0.34 to 0.23)	-0.12<br>(-0.43 to 0.14)	-0.32<br>(-0.61 to -0.04)	0.01<br>(-0.40 to 0.25)	-0.23<br>(-0.49 to 0.10)	-0.23<br>(-0.56 to 0.13)	-0.41<br>(-0.67 to -0.13)	-0.07<br>(-0.49 to 0.27)	-0.27<br>(-0.53 to 0.06)
0-150ms RFD (N·s^{-1})	-0.04<br>(-0.63 to 0.30)	-0.32<br>(-0.67 to -0.03)	0.06<br>(-0.36 to 0.31)	-0.03<br>(-0.38 to 0.31)	-0.09<br>(-0.54 to 0.30)	-0.39<br>(-0.72 to 0.01)	0.03<br>(-0.47 to 0.31)	-0.26<br>(-0.53 to 0.07)	-0.24<br>(-0.68 to 0.27)	**-0.64***<br>**(-0.78 to -0.38)**	-0.19<br>(-0.65 to 0.18)	-0.29<br>(-0.59 to 0.17)
0-200ms RFD (N·s^{-1})	-0.01<br>(-0.53 to 0.27)	-0.25<br>(-0.60 to 0.08)	0.09<br>(-0.40 to 0.43)	-0.03<br>(-0.36 to 0.35)	-0.030<br>(-0.44 to 0.29)	-0.32<br>(-0.67 to 0.11)	0.09<br>(-0.41 to 0.40)	-0.18<br>(-0.49 to 0.19)	-0.17<br>(-0.56 to 0.25)	**-0.56**<br>**(-0.75 to -0.23)**	-0.13<br>(-0.60 to 0.28)	-0.22<br>(-0.51 to 0.18)
0-250ms RFD (N·s^{-1})	0.05<br>(-0.48 to 0.29)	-0.22<br>(-0.61 to 0.16)	0.08<br>(-0.48 to 0.46)	-0.08<br>(-0.37 to 0.30)	0.03<br>(-0.39 to 0.30)	-0.26<br>(-0.67 to 0.19)	0.09<br>(-0.43 to 0.47)	-0.11<br>(-0.39 to 0.23)	-0.07<br>(-0.45 to 0.25)	-0.46<br>(-0.74 to -0.11)	-0.10<br>(-0.54 to 0.32)	-0.20<br>(-0.46 to 0.09)

Table displaying the relationships at 10, 12, and 14 km·h−1. Values in brackets represent 95% bootstrap confidence intervals. Data were analyzed using Pearson correlation coefficients (n = 23 participants). *indicates statistical significance ($p < 0.05$). Bold font indicates $|r| \geq 0.55$. Abbreviations: iEMG, integrated EMG; GM, gastrocnemius medialis; GL, gastrocnemius lateralis; SOL, soleus; TA, tibialis anterior; PF, relative peak force; RFD, rate of force development.

Table S5. Correlation coefficients between knee joint strength at 60°·s^{-1} and knee muscles' CNA at 10, 12, and 14 km·h^{-1}. Related to Figure 1.

	VM CNA at 10 km·h^{-1}	VL CNA at 10 km·h^{-1}	RF CNA at 10 km·h^{-1}	BF CNA at 10 km·h^{-1}	VM CNA at 12 km·h^{-1}	VL CNA at 12 km·h^{-1}	RF CNA at 12 km·h^{-1}	BF CNA at 12 km·h^{-1}	VM CNA at 14 km·h^{-1}	VL CNA at 14 km·h^{-1}	RF CNA at 14 km·h^{-1}	BF CNA at 14 km·h^{-1}
$K_{flex-con}$ at 60°s^{-1} (Nm·kg^{-1} FFM)	-0.28 (-0.54 to 0.25)	-0.17 (-0.61 to 0.26)	-0.24 (-0.66 to 0.21)	-0.44 (-0.85 to 0.07)	-0.53* (-0.79 to 0.20)	-0.13 (-0.45 to 0.19)	-0.36 (-0.70 to -0.01)	**-0.56* (-0.82 to -0.16)**	-0.37 (-0.64 to 0.12)	-0.07 (-0.41 to 0.30)	-0.24 (-0.54 to 0.11)	-0.22 (-0.56 to 0.17)
K_{ex-con} at 60°s^{-1} (Nm·kg^{-1} FFM)	-0.05 (-0.47 to 0.42)	-0.07 (-0.41 to 0.36)	-0.24 (-0.53 to 0.23)	-0.11 (-0.35 to 0.26)	-0.11 (-0.42 to 0.36)	-0.13 (-0.49 to 0.34)	-0.01 (-0.39 to 0.30)	-0.02 (-0.31 to 0.25)	-0.29 (-0.61 to 0.20)	0.00 (-0.39 to 0.38)	-0.05 (-0.50 to 0.23)	-0.05 (-0.45 to 0.42)
$K_{flex-ecc}$ at 60°s^{-1} (Nm·kg^{-1} FFM)	-0.26 (-0.59 to 0.09)	-0.38 (-0.67 to 0.03)	-0.35 (-0.65 to 0.11)	-0.57 (-0.76 to -0.27)	-0.30 (-0.61 to 0.03)	-0.43 (-0.70 to -0.08)	-0.33 (-0.62 to 0.15)	**-0.62* (-0.79 to -0.43)**	-0.34 (-0.65 to 0.00)	-0.49 (-0.69 to -0.24)	-0.42 (-0.64 to 0.05)	**-0.63* (-0.81 to -0.31)**
K_{ex-ecc} at 60°s^{-1} (Nm·kg^{-1} FFM)	0.13 (-0.40 to 0.58)	-0.12 (-0.51 to 0.27)	-0.14 (-0.48 to 0.29)	-0.20 (-0.48 to 0.13)	-0.06 (-0.44 to 0.40)	-0.18 (-0.53 to 0.14)	-0.32 (-0.59 to 0.19)	-0.40 (-0.68 to -0.03)	-0.13 (-0.57 to 0.38)	-0.25 (-0.57 to 0.12)	-0.37 (-0.65 to 0.17)	-0.40 (-0.75 to 0.15)

Table displaying the relationships at 10, 12, and 14 km·h−1. Values in brackets represent 95% bootstrap confidence intervals. Data were analyzed using Pearson correlation coefficients (n = 23 participants). *indicates statistical significance ($p < 0.05$). Bold font indicates $|r| \geq 0.55$. Abbreviations: CNA, cumulative neuromuscular activation; FFM, fat-free mass; VM, vastus medialis; VL, vastus lateralis; RF, rectus femoris; BF, biceps femoris; Kflex-con, knee flexor muscles relative peak torque in concentric action; Kex-con, knee extensor muscles relative peak torque in concentric action; Kflex-ecc, knee flexor muscles relative peak torque in eccentric action; Kex-ecc, knee extensor muscles relative peak torque in eccentric action.

Table S6. Correlation coefficients between ankle joint strength at 60°·s^{-1} and ankle muscles' CNA at 10, 12, and 14 km·h^{-1}. Related to Figure 1.

	GM CNA at 10 km·h^{-1}	GL CNA at 10 km·h^{-1}	SOL CNA at 10 km·h^{-1}	TA CNA at 10 km·h^{-1}	GM CNA at 12 km·h^{-1}	GL CNA at 12 km·h^{-1}	SOL CNA at 12 km·h^{-1}	TA CNA at 12 km·h^{-1}	GM CNA at 14 km·h^{-1}	GL CNA at 14 km·h^{-1}	SOL CNA at 14 km·h^{-1}	TA CNA at 14 km·h^{-1}
$A_{dors-con}$ at 60°s^{-1} (Nm·kg^{-1} FFM)	0.12 (-0.15 to 0.49)	0.00 (-0.34 to 0.33)	0.01 (-0.21 to 0.25)	-0.14 (-0.48 to 0.17)	0.10 (-0.18 to 0.42)	0.04 (-0.27 to 0.38)	-0.09 (-0.42 to 0.29)	-0.07 (-0.45 to 0.32)	0.18 (-0.15 to 0.52)	0.02 (-0.29 to 0.40)	-0.15 (-0.51 to 0.25)	-0.11 (-0.41 to 0.20)
$A_{plan-con}$ at 60°s^{-1} (Nm·kg^{-1} FFM)	0.28 (-0.35 to 0.61)	-0.20 (-0.70 to 0.30)	0.29 (-0.37 to 0.63)	0.13 (-0.20 to 0.39)	0.28 (-0.30 to 0.62)	-0.16 (-0.65 to 0.27)	0.31 (-0.37 to 0.62)	-0.06 (-0.37 to 0.23)	0.26 (-0.21 to 0.62)	-0.43 (-0.70 to -0.19)	0.20 (-0.30 to 0.55)	-0.20 (-0.48 to 0.08)
$A_{dors-ecc}$ at 60°s^{-1} (Nm·kg^{-1} FFM)	0.15 (-0.30 to 0.46)	-0.23 (-0.50 to 0.06)	-0.01 (-0.32 to 0.19)	-0.23 (-0.46 to 0.00)	0.18 (-0.30 to 0.52)	-0.11 (-0.38 to 0.18)	0.01 (-0.34 to 0.21)	-0.11 (-0.32 to 0.16)	0.26 (-0.23 to 0.64)	-0.17 (-0.44 to 0.17)	-0.13 (-0.38 to 0.15)	-0.28 (-0.53 to 0.02)
$A_{plan-ecc}$ at 60°s^{-1} (Nm·kg^{-1} FFM)	0.11 (-0.31 to 0.38)	-0.36 (-0.69 to 0.00)	0.05 (-0.36 to 0.41)	0.08 (-0.21 to 0.40)	0.13 (-0.24 to 0.42)	-0.31 (-0.62 to 0.04)	0.06 (-0.35 to 0.32)	0.13 (-0.14 to 0.45)	0.23 (-0.14 to 0.53)	-0.36 (-0.63 to -0.07)	0.00 (-0.32 to 0.31)	-0.09 (-0.40 to 0.27)

Table displaying the relationships at 10, 12, and 14 km·h−1. Values in brackets represent 95% bootstrap confidence intervals. Data were analyzed using Pearson correlation coefficients (n = 23 participants). Abbreviations: CNA, cumulative neuromuscular activation; FFM, fat-free mass; GM, gastrocnemius medialis; GL, gastrocnemius lateralis; SOL, soleus; TA, tibialis anterior; Adors-con, dorsiflexor muscles relative peak torque in concentric action; Aplan-con, plantar flexor muscles relative peak torque in concentric action; Adors-ecc, dorsiflexor muscles relative peak torque in eccentric action; Aplan-ecc, plantar flexor muscles relative peak torque in eccentric action.

Table S7. Correlation coefficients betweenIsometric Force-Time Characteristics and knee muscles' CNA at 10, 12, and 14 km·h^{-1}. Related to Figure 3.

	VM CNA at 10 km·h^{-1}	VL CNA at 10 km·h^{-1}	RF CNA at 10 km·h^{-1}	BF CNA at 10 km·h^{-1}	VM CNA at 12 km·h^{-1}	VL CNA at 12 km·h^{-1}	RF CNA at 12 km·h^{-1}	BF CNA at 12 km·h^{-1}	VM CNA at 14 km·h^{-1}	VL CNA at 14 km·h^{-1}	RF CNA at 14 km·h^{-1}	BF CNA at 14 km·h^{-1}
PF (N·kg^{-1} FFM)	0.23 (-0.31 to 0.66)	-0.27 (-0.55 to 0.08)	-0.09 (-0.50 to 0.43)	-0.14 (-0.45 to 0.22)	0.04 (-0.35 to 0.46)	-0.31 (-0.59 to -0.05)	-0.12 (-0.45 to 0.46)	-0.22 (-0.51 to 0.11)	0.04 (-0.39 to 0.54)	0.05 (-0.60 to 0.25)	-0.06 (-0.39 to 0.50)	-0.20 (-0.50 to 0.16)
0-50ms RFD (N·s^{-1})	0.31 (-0.27 to 0.72)	0.16 (-0.17 to 0.59)	-0.01 (-0.37 to 0.36)	-0.03 (-0.32 to 0.39)	0.22 (-0.18 to 0.62)	0.26 (-0.09 to 0.60)	-0.02 (-0.43 to 0.24)	-0.04 (-0.39 to 0.25)	0.24 (-0.30 to 0.66)	-0.01 (-0.17 to 0.47)	0.04 (-0.31 to 0.32)	0.05 (-0.29 to 0.40)
0-100ms RFD(N·s^{-1})	0.59 (0.27 to 0.81)	0.20 (-0.12 to 0.60)	0.13 (-0.21 to 0.48)	0.04 (-0.25 to 0.32)	0.31 (0.01 to 0.61)	0.19 (-0.09 to 0.51)	0.13 (-0.13 to 0.39)	0.07 (-0.22 to 0.35)	0.38 (0.08 to 0.66)	-0.05 (-0.24 to 0.32)	0.13 (-0.15 to 0.47)	-0.07 (-0.40 to 0.29)
0-150ms RFD(N·s^{-1})	0.39 (0.06 to 0.68)	0.16 (-0.21 to 0.60)	0.23 (-0.03 to 0.52)	0.18 (-0.11 to 0.49)	0.20 (-0.12 to 0.49)	0.14 (-0.18 to 0.48)	0.35 (-0.10 to 0.58)	0.21 (-0.29 to 0.54)	0.19 (-0.15 to 0.54)	0.02 (-0.32 to 0.24)	0.38 (-0.14 to 0.64)	0.02 (-0.46 to 0.54)
0-200ms RFD(N·s^{-1})	0.23 (-0.22 to 0.68)	-0.01 (-0.36 to 0.35)	0.25 (0.01 to 0.55)	0.12 (-0.17 to 0.41)	0.08 (-0.28 to 0.46)	-0.08 (-0.38 to 0.27)	0.19 (-0.14 to 0.43)	-0.01 (-0.46 to 0.31)	0.05 (-0.33 to 0.48)	-0.02 (-0.52 to 0.12)	0.26 (-0.01 to 0.51)	-0.22 (-0.59 to 0.21)
0-250ms RFD(N·s^{-1})	0.24 (-0.23 to 0.69)	-0.05 (-0.38 to 0.31)	0.28 (0.00 to 0.59)	0.08 (-0.19 to 0.40)	0.06 (-0.31 to 0.44)	-0.14 (-0.43 to 0.17)	0.09 (-0.15 to 0.43)	-0.12 (-0.48 to 0.18)	0.05 (-0.34 to 0.52)	0.03 (-0.58 to 0.21)	0.13 (-0.09 to 0.58)	-0.35 (-0.67 to 0.04)

Table displaying the relationships at 10, 12, and 14 km·h−1. Values in brackets represent 95% bootstrap confidence intervals. Data were analyzed using Pearson correlation coefficients (n = 23 participants). Abbreviations: CNA, cumulative neuromuscular activation; FFM, fat-free mass; VM, vastus medialis; VL, vastus lateralis; RF, rectus femoris; BF, biceps femoris; PF, relative peak force; RFD, rate of force development.

Table S8. Correlation coefficients betweenIsometric Force-Time Characteristics and ankle muscles' CNA at 10, 12, and 14 km·h^{-1}. Related to Figure 3.

	GM CNA at 10 km·h^{-1}	GL CNA at 10 km·h^{-1}	SOL CNA at 10 km·h^{-1}	TA CNA at 10 km·h^{-1}	GM CNA at 12 km·h^{-1}	GL CNA at 12 km·h^{-1}	SOL CNA at 12 km·h^{-1}	TA CNA at 12 km·h^{-1}	GM CNA at 14 km·h^{-1}	GL CNA at 14 km·h^{-1}	SOL CNA at 14 km·h^{-1}	TA CNA at 14 km·h^{-1}
PF (N·kg^{-1} FFM)	0.11 (-0.35 to 0.39)	-0.14 (-0.42 to 0.15)	0.20 (0.01 to 0.44)	0.19 (-0.16 to 0.54)	0.09 (-0.39 to 0.39)	-0.15 (-0.48 to 0.16)	0.10 (-0.26 to 0.34)	-0.01 (-0.32 to 0.32)	0.02 (-0.46 to 0.45)	-0.23 (-0.60 to 0.09)	-0.06 (-0.40 to 0.27)	-0.22 (-0.59 to 0.13)
0-50ms RFD (N·s^{-1})	-0.16 (-0.36 to 0.08)	-0.58 (-0.77 to -0.38)	-0.24 (-0.52 to 0.05)	0.04 (-0.43 to 0.49)	-0.09 (-0.31 to 0.25)	-0.51 (-0.72 to -0.21)	-0.15 (-0.53 to 0.21)	-0.25 (-0.61 to 0.23)	-0.14 (-0.42 to 0.25)	-0.49 (-0.70 to -0.18)	-0.17 (-0.59 to 0.33)	-0.22 (-0.57 to 0.27)
0-100ms RFD(N·s^{-1})	-0.09 (-0.45 to 0.12)	-0.37 (-0.62 to -0.15)	-0.03 (-0.34 to 0.16)	-0.09 (-0.34 to 0.19)	-0.12 (-0.45 to 0.13)	-0.32 (-0.60 to -0.08)	0.01 (-0.31 to 0.25)	-0.23 (-0.50 to 0.06)	-0.22 (-0.54 to 0.14)	-0.40 (-0.64 to -0.12)	-0.05 (-0.44 to 0.30)	-0.25 (-0.50 to 0.09)
0-150ms RFD(N·s^{-1})	-0.07 (-0.63 to 0.26)	-0.36 (-0.69 to -0.04)	0.02 (-0.40 to 0.27)	-0.05 (-0.37 to 0.28)	-0.10 (-0.58 to 0.27)	-0.41 (-0.71 to -0.11)	0.02 (-0.48 to 0.28)	-0.28 (-0.52 to 0.02)	-0.24 (-0.64 to 0.28)	**-0.63* (-0.77 to -0.42)**	-0.20 (-0.64 to 0.19)	-0.28 (-0.57 to 0.12)
0-200ms RFD(N·s^{-1})	-0.05 (-0.55 to 0.23)	-0.28 (-0.62 to 0.08)	0.05 (-0.42 to 0.38)	-0.06 (-0.40 to 0.30)	-0.05 (-0.45 to 0.24)	-0.34 (-0.68 to 0.00)	0.07 (-0.45 to 0.37)	-0.19 (-0.48 to 0.18)	-0.18 (-0.52 to 0.20)	**-0.58* (-0.77 to -0.27)**	-0.17 (-0.65 to 0.28)	-0.22 (-0.49 to 0.12)
0-250ms RFD(N·s^{-1})	0.03 (-0.54 to 0.29)	-0.25 (-0.63 to 0.16)	0.04 (-0.49 to 0.44)	-0.11 (-0.39 to 0.29)	0.01 (-0.39 to 0.25)	-0.28 (-0.66 to 0.11)	0.07 (-0.47 to 0.43)	-0.12 (-0.40 to 0.19)	-0.08 (-0.45 to 0.25)	-0.48 (-0.75 to -0.14)	-0.13 (-0.60 to 0.35)	-0.20 (-0.42 to 0.05)

Table displaying the relationships at 10, 12, and 14 km·h−1. Values in brackets represent 95% bootstrap confidence intervals. Data were analyzed using Pearson correlation coefficients (n = 23 participants). *indicates statistical significance ($p < 0.05$). Bold font indicates $|r| \geq 0.55$. Abbreviations: CNA, cumulative neuromuscular activation; FFM, fat-free mass; GM, gastrocnemius medialis; GL, gastrocnemius lateralis; SOL, soleus; TA, tibialis anterior; PF, relative peak force; RFD, rate of force development.
